# NT-pro BNP in AECOPD-PH: old biomarker, new insights-based on a large retrospective case-controlled study

**DOI:** 10.1186/s12931-021-01917-3

**Published:** 2021-12-27

**Authors:** Fengming Tian, Wen Song, Liang Wang, Qiang Zeng, Zhenyu Zhao, Ning Feng, Jiahui Fan, Yue Wang, Jing Wang, Xiumin Ma

**Affiliations:** 1 State Key Laboratory of Pathogenesis, Prevention and Treatment of High Incidence Diseases in Central Asia, Clinical Laboratory Center, Tumor Hospital Affiliated to Xinjiang Medical University, No. 789 Suzhou Road, Urumqi, 830011 Xinjiang Uygur Autonomous Region People’s Republic of China; 2 First Affiliated Hospital of Xinjiang Medical University, Urumqi, Xinjiang 830011 People’s Republic of China; 3 Clinical Laboratory Center, Affiliated Traditional Chinese Medicine Hospital of Xinjiang Medical University, Urumqi, Xinjiang 830099 People’s Republic of China; 4 School of Stomatology, Jilin University, Changchun, Jilin, 130021 People’s Republic of China; 5 Respiratory Department of the second affiliated Hospital of Hainan Medical College, Haikou, Hainan 570000 People’s Republic of China

**Keywords:** Acute exacerbations of chronic obstructive pulmonary disease, Pulmonary hypertension, NT-proBNP

## Abstract

**Background:**

Pulmonary hypertension (PH) is one of the common complications in chronic obstructive pulmonary disease (COPD). The study aimed to evaluate the predicting ability of N-terminal pro brain natriuretic peptide (NT-pro BNP) in patients with AECOPD-PH and its relationship with the severity of PH.

**Methods:**

A large retrospective case-controlled study (n = 1072) was performed in the First Affiliated Hospital of Xinjiang Medical University from January 2018 to December 2020, and patients were divided into stable COPD (n = 178), AECOPD (n = 688) and AECOPD-PH group (n = 206). Different statistical models were used to screen for reliable and stable biomarkers.

**Results:**

In unadjusted analysis and PSM (model 1, 2, 3), red cell distribution width (RDW), total bilirubin (TBIL), and NT-pro BNP were higher in patients with AECOPD-PH than those in AECOPD group. Logistic regression analysis showed, when the range of NT-proBNP was 271–1165 pg/mL (OR: 0.293; 95%CI: 0.184–0.467; P < 0.001) and NT-proBNP > 1165 pg/mL (OR: 0.559; 95%CI: 0.338–0.926; P = 0.024), the morbidity risk of PH in AECOPD patients was increased, so did TBIL. In receiver operating characteristic (ROC) curves, at the cut-off value of NT-proBNP was 175.14 pg/mL, AUC was 0.651 (P < 0.001), which was better than TBIL (AUC: 0.590, P < 0.001). As for the results of rank correlation analysis, NT-proBNP had a weak correlation with severity of PH with AECOPD (*r*_*s*_ = 0.299, P = 0.001) and its relative relevance with other biomarkers (RDW was 0.359 and TBIL was 0.238, P < 0.001).

**Conclusions:**

Our findings suggest that NT-proBNP has a diagnostic efficacy in AECOPD-PH and NT-proBNP has a weak correlation with severity of PH with AECOPD.

## Background

Pulmonary hypertension (PH) may occur in patients with chronic obstructive pulmonary disease (COPD) due to inadequate alveolar ventilation, hypoxia, and pulmonary vascular remodeling [1, 2]. A recently study found that approximately 1% of the world’s population has PH and that the prevalence among people over 65 years old increases to 10% [1]. One third of deaths in COPD patients relate to cardiovascular disease, equaling or exceeding pulmonary related disease mortality [3]. However, it may be early diagnosis can be difficult to distinguish between COPD patients with acute exacerbations alone or acute exacerbations with PH. COPD exacerbations (AECOPD) are episodes of increased respiratory symptoms, particularly dyspnoea, cough and sputum [4]. PH symptoms such as dyspnea on exertion, shortness of breath, and fatigue are non-specific [5].

N-terminal pro brain natriuretic peptide (NT-proBNP) is secreted by cardiomyocytes in response to ventricular stretch and is a noninvasive marker of right ventricular dysfunction [6]. NT-proBNP levels correlate with functional capacity, right ventricular function, echocardiographic and hemodynamic variables. It plays an important role in maintaining cardiopulmonary homeostasis. Serum concentrations of NT-proBNP are elevated in clinical conditions that affect not only left (LV) but also right ventricle (RV), both markers can be used as prognostic parameters for PH and have been recommended in the current guidelines [7, 8]. Management and treatment of cardiovascular disease in patients with COPD is critical to reduce morbidity and mortality [9]. A recently study showed that the low, medium, and high NT-proBNP categories as part of the multiparametric risk assessment approach included in the PH patient management scope [7]. The level of NT-proBNP is highly prognostic of PH progression, and patients with persistently high NT-proBNP may have the highest risk of disease progression [6, 10]. NT-proBNP is powerful independent predictors of death and adverse events in heart failure, a broad range of cardiovascular conditions even in asymptomatic individuals.

Current research suggested that timely intensive management of those with a raised NT-proBNP detected may be useful in identifying cardiovascular disease, stratifying risk, and guiding the treatment of COPD [7]. These cardiovascular treatments could reduce the incidence of heart failure in patients [9]. However, chest tightness, shortness of breath, cough and sputum may only be acute exacerbation of COPD, or may be caused by COPD complicated with PH. We therefore undertook retrospective analysis study to direct future research and provided evidence. The study aims to define the associations and diagnostic accuracy of NT-proBNP elevation patients in AECOPD complicated with PH.

## Methods

### Patients

A single center cross-sectional study was performed at First Affiliated Hospital of Xinjiang Medical University from January 2018 to December 2020, patients were divided into stable COPD, AECOPD and AECOPD patients complicated with PH (AECOPD-PH). The research was approved by Xinjiang Medical University Ethical Committees (Approved number: 20170214-50) and all patients provided written informed consent. The enrolled patients were diagnosed as regarding GOLD criteria [11]. The patients seemed to have PH (PASP > 30 mmHg), and were classified into three subgroups, mild (30–50 mmHg), moderate (50–70 mmHg), and severe (> 70 mmHg) degrees [2].

### Data collection

All data was obtained. Patients’ blood pressure and pulse rate measured on admission; BMI, smoking status, complications, biomarkers: white blood cell (WBC), red blood cell (RBC), red cell distribution width (RDW), mean volume (MPV), fibrinogen degradation products (FDP), D-Dimer (DD), albumin globulin ratio (A/G), total bilirubin (TBIL), NT-proBNP and so on. The echocardiography results of patients in the AECOPD-PH group were collected.

### Exclusion criteria

**Fig. 1:**
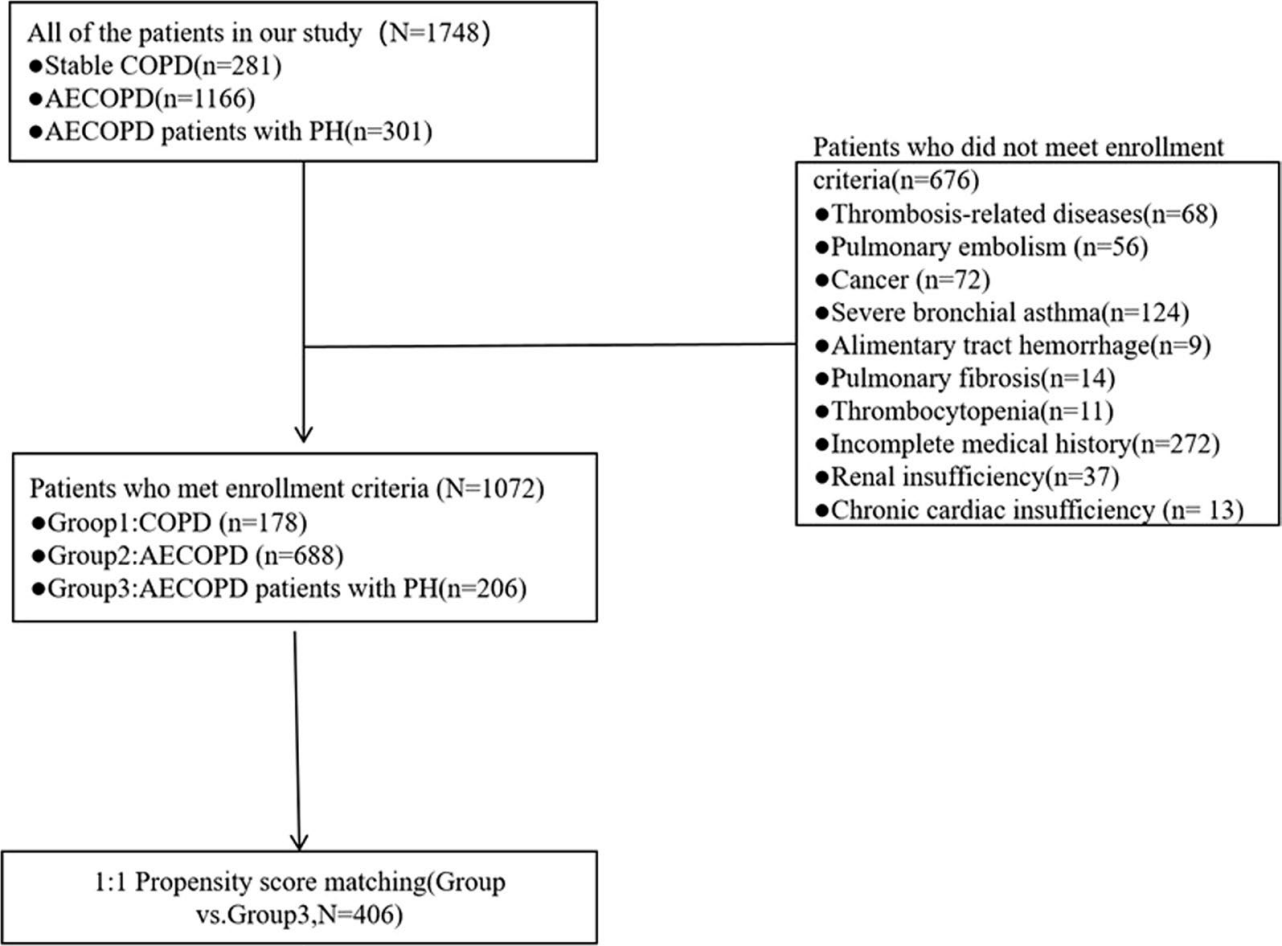
Exclusions criteria

Patients underwent thorough pulmonary and cardiologic preinclusion screening, and those with pulmonary disease other than COPD, as well as chronic cardiac insufficiency were excluded. This study included the number of 1072 patients; 676 patients were excluded (details shown in Fig. 1). Patients were divided into three groups, Group 1—COPD group (n = 178); Group 2—AECOPD group (n = 688); Group 3—AECOPD-PH group (n = 206).

### Biochemical and echocardiographic test

WBC, RBC, RDW, and MPV (XN-2000, SYSMEX, Japan). ALB, TG, TC, HDL, LDL and TBIL (Cobas8000, Roche, Germany). FDP and DD (ACL-TOP-750, Werfen, Spain). NT-ProBNP (VITROS 5600, Ortho Clinical Diagnostics, American). CRP (VITROS 5600, Ortho Clinical Diagnostics, American), IL-6 and PCT (Cobas701, Roche, Germany). Echocardiography was performed in AECOPD-PH patients (EPIQ-7C, Philips, Netherlands).

### Statistical analysis

All analyses were performed by SPSS 25 (IBM, American). Quantitative variables were described as median (interquartile range, IQR) and the Wilcoxon rank-sum test was used to compare between groups, expressed as mean ± standard deviation, and *t* test was used to compare between groups. Qualitative variables were described as frequency (number, percentages) and compared between two groups by the Fisher exact test or the Chi-square test as appropriate. To reduce selection bias for treatment and any other related potential confounding factor, we performed a baseline characteristic adjustment for patients by propensity matching score (PSM). PSM details showed in Table 2 (model 1, 2 and 3, matched 1:1, calipers value = 0.05). Then the markers were layered, stepwise multivariate Logistic regression was used to calculate the adjusted OR and its 95%CI. The diagnostic performance of biomarkers was statistically significant in the above statistical analysis by means of receiver operating characteristic (ROC) curves, the optimal threshold on the curve was considered to predict AECOPD-PH. Finally, rank correlation analysis was used to analyze the correlation between NT-ProBNP and the severity of AECOPD-PH, and the relative relevance of NT-ProBNP with RDW / TBIL. P value < 0.05 was significant for statistical analyses.

## Results

### Demographics and clinical presentations

Overall, among 1748 patients included, finally 1702 patients were enrolled into this study (Fig. 1). Median age was 73 years old (IQR 65–80), 440 patients (25.85%) were female. The pulse rate of AECOPD patients was higher than that of COPD group. Among the patients, there were 134 (12.50%) patients still smoking, and 242 (22.57%) were ex-smokers. There were 235 (21.92%) patients complicated with diabetes mellitus, 637 (59.14%) patients complicated with coronary atherosclerotic heart disease, it was almost as many patients as complicated with hypertension (n = 634, 59.14%). 20.43% (n = 219) of patients had a history of cerebral infarction. Most patients with AECOPD-PH have mild or moderate pulmonary hypertension (n = 156, 75.73%) (Table 1).

**Table 1:** General demographic characteristics of case and control

Variables	Group 1	Group 2	Group 3	All groups	P value		
	(n = 178)	(n = 688)	(n = 206)		1 vs 2	1 vs 3	2 vs 3
Age (year)	68 (61–77)	75 (66–81)	73 (65–81)	< 0.001	< 0.001	< 0.001	0.171
Sex (M/F)	101 (56.74%)/77 (43.26%)	430 (62.50%)/258 (37.50%)	101 (49.03%)/105 (50.97%)	0.002	0.16	0.131	0.001
Systolic blood pressure (mmHg)	129.58 ± 16.97	130.85 ± 20.28	128.00 ± 17.77	0.13	0.094	0.452	0.06
Diastolic blood pressure (mmHg)	75.38 ± 10.99	76.60 ± 12.54	76.46 ± 12.21	0.516	0.386	0.996	0.355
Pulse rate (PR, beats /per minute)	80 ± 14	85 ± 14	87 ± 14	< 0.001	< 0.001	< 0.001	0.498
Body Mass Index (BMI, kg/m^2^)	25 (23–28)	25 (22–28)	24 (21–28)	0.097	0.97	0.38	0.319
*Smoking status (n, SD/%)*							
Smoking index (SI)	284.09 ± 496.40	223.27 ± 399.29	172.74 ± 346.47	0.41	0.436	0.19	0.028
Smoker	34 (19.10%)	88 (12.79%)	12 (5.83%)	0.004	0.031	< 0.001	0.005
Ex-smoker	35 (19.66%)	162 (23.55%)	45 (21.84%)	0.523	0.271	0.6	0.611
length of stay (LOS)	7 (6–8)	8 (7–11)	8 (7–10)	< 0.001	< 0.001	< 0.001	0.606
*Complications*							
Diabetes mellitus (DM)	36 (20.22%)	158 (22.97%)	41 (19.90%)	0.541	0.434	0.937	0.354
Coronary atherosclerotic heart disease (CHD)	46 (25.84%)	530 (77.03%)	61 (29.61%)	< 0.001	< 0.001	0.411	< 0.001
Hypertension	88 (49.43%)	404 (58.72%)	142 (68.93%)	< 0.001	0.026	< 0.001	0.008
History of cerebral infarction	39 (21.91%)	153 (22.24%)	27 (13.17%)	0.015	0.925	0.023	0.004
Procalcitonin (PCT, ng/mL)	0.03 (0.01–0.05)	0.05 (0.03–0.09)	0.05 (0.03–0.08)	< 0.001	< 0.001	< 0.001	0.752
C-reactive protein (CRP, mg/L)	3.60 (1.57–6.54)	11.69 (4.08–36.28)	9.71 (3.50–25.30)	< 0.001	< 0.001	< 0.001	0.092
Interleukin 6 (IL-6, pg/mL)	4.93 (2.18–9.73)	9.52 (3.67–30.48)	8.54 (3.44–21.65)	< 0.001	< 0.001	< 0.001	0.7
Hemoglobin (g/L)	140 (128–150)	114 (118–144)	134 (116–150)	< 0.001	< 0.001	< 0.001	0.147
White blood cell count (WBC, × 10^9^ /L)	6.43 (5.65–7.84)	7.15 (5.83–9.06)	6.84 (5.41–8.34)	< 0.001	< 0.001	0.11	0.013
Red blood cell count (RBC, × 10^12^ /L)	4.64 (4.21–4.97)	3.96 (4.41–4.85)	4.45 (3.98–5.04)	< 0.001	< 0.001	0.434	0.4
Red cell Ddistribution width (RDW, %)	13.2 (12.7–13.8)	13.5 (12.9–14.6)	13.9 (13.2–15.5)	< 0.001	0.001	0.164	< 0.001
Mean platelet volume (MPV, fL)	11.4 (10.1–12.9)	10.9 (10.0–12.6)	11.3 (10.3–12.8)	0.086	0.109	0.999	0.065
Fibrinogen degrdtion products (FDP, μg/mL)	1.01 (0.65–1.64)	1.83 (1.08–3.52)	2.05 (1.14–3.28)	< 0.001	< 0.001	< 0.001	0.499
D-Dimer (DD, ng/mL)	142 (85–241)	250 (148–488)	264 (154–501)	< 0.001	< 0.001	< 0.001	0.743
Albumin (ALB, g/L)	40.2 (37.7–43.8)	37.3 (33.6–41.1)	37.1 (33.2–40.7)	< 0.001	< 0.001	< 0.001	0.513
Abnormal albumin globulin ratio (A/G)	1.31 (1.13–1.56)	1.20 (1.06–1.39)	1.17 (1.03–1.348)	< 0.001	< 0.001	< 0.001	0.091
Triglyceride (TG, mmol/L)	1.35 (1.05–1.93)	1.12 (0.81–1.57)	1.08 (0.79–1.50)	< 0.001	< 0.001	< 0.001	0.41
Total cholesterol (TC, mmol/L)	3.96 (3.43–4.61)	3.63 (3.04–4.28)	3.66 (3.04–4.33)	< 0.001	< 0.001	0.001	0.842
High density lipoprotein cholesterol l(HDL-C, mmol/L)	1.03 (0.86–1.26)	1.04 (0.83–1.30)	1.04 (0.81–1.31)	0.915	0.701	0.683	0.994
Low density lipoprotein cholesterol (LDL-C, mmol/L)	2.58 (2.01–3.15)	2.27 (1.77–2.84)	2.26 (1.74–2.91)	0.007	< 0.001	0.009	0.841
Total bilirubin (TBIL, μmol/L)	11.2 (8.4–14.7)	11.5 (8.8–15.5)	13.2 (9.5–18.8)	< 0.001	0.473	< 0.001	< 0.001
N terminal pro B type natriuretic peptide (NT-proBNP, pg/mL)		93.4 (3.59–316.69)	273.53 (83.31–1023.70)				< 0.001
Mild (n, %)			75 (36.41%)				
Moderate (n, %)			81 (39.32%)				
Severe (n, %)			50 (24.27%)				
Pulmonary artery systolic pressure (PASP, mmHg)			61 (48–76)				
Left ventricular end-diastolic volume (LEDV, mL)			46.57 ± 4.45				
Left ventricular ejection fraction (LVEF, %)			61.01 ± 5.76				
Pulmonary artery internal dimension (PAD, mm)			28.92 ± 5.40				

### Blood biomarkers

**Fig. 2:**
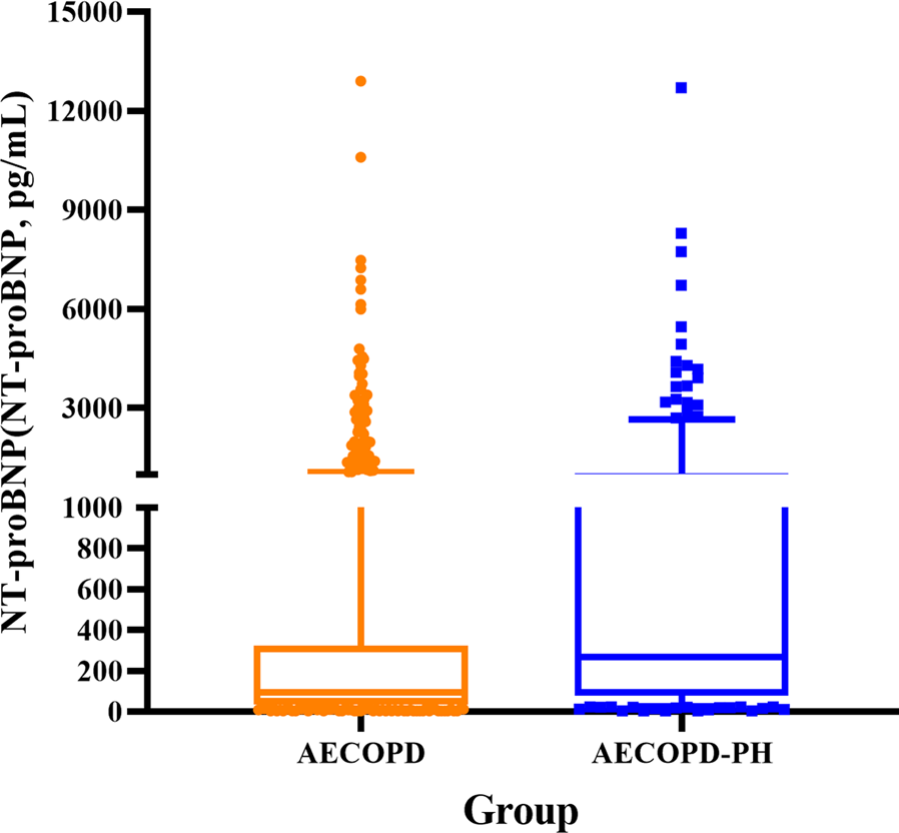
Comparison of NT-proBNP levels between AECOPD group and AECOPD-PH group

PCT, CRP and IL-6 had significant differences in three groups (P < 0.001), however there was no difference between AECOPD-PH group and AECOPD group, so did WBC, Hb, MPV, DD, FDP, ALB, A/G, TG, TC and HDL. Among the three groups, RDW in AECOPD-PH group (13.9, 13.2–15.5%) was higher than that in AECOPD group (13.5, 12.9–14.6%) and COPD group (13.2, 12.7–13.8%), but there was no difference between COPD and AECOPD-PH group (P = 0.164). The serum TBIL level of AECOPD-PH group (13.2, 9.5–18.8 μmol/L) was not only higher than that of AECOPD group (11.8, 8.8–15.5 μmol/L), but also higher than COPD group (11.2, 8.4–14.7 μmol/L). However, there was no significant difference between COPD and AECOPD groups (P = 0.473). The level of NT-proBNP in AECOPD-PH group (273.53, 83.31–1023.70 pg/mL) was significantly higher than that of AECOPD group (93.4, 3.59–316.69 pg/mL, P < 0.001). Since most COPD patients were admitted to the hospital for other diseases, there were few patients who need to complete the NT-proBNP examination, so statistical analysis is not done here (Table 1 and Fig. 2).

### Propensity score matching

**Fig. 3:**
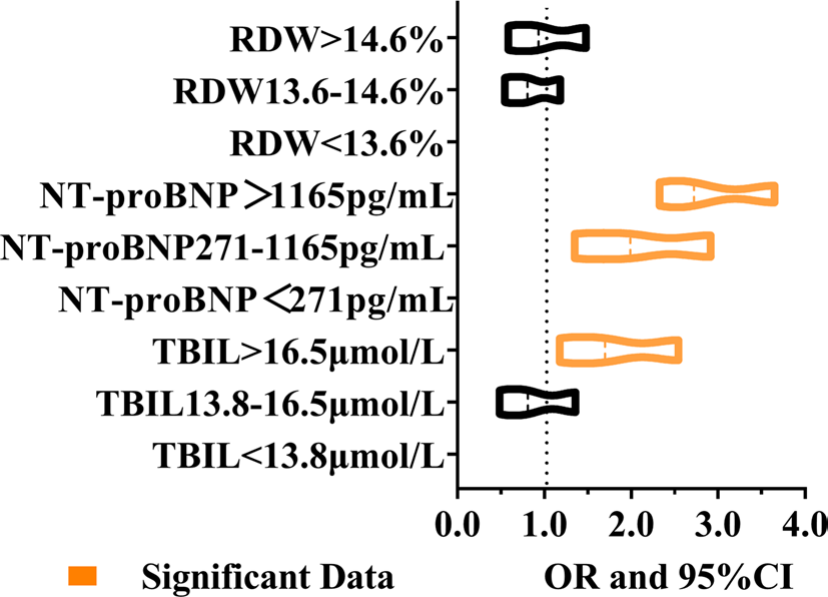
RDW, TBIL and NT-proBNP were stratified, and multivariate logistic regression was performed to investigate associations between biomarkers levels and the prediction of AECOPD-PH

Many variables were statistically different. The matched variables showed no difference between AECOPD and AECOPD-PH group after PSM. 206 patients in each cohort were compared (Table 2 and Fig. 3). After three PSM models, WBC, RDW, TBIL and NT-proBNP still had statistical difference. However, the increased WBC was non-specific, so no further analysis was performed. Therefore, in follow-up studies, we analyzed the clinical significance of NT-pBNP, TBIL and RDW in AECOPD-PH patients.

**Table 2:** Propensity matching scoring model (n = 412)

Variables	P value (Before PSM, n = 412)	P value (After PSM, n = 412)		
		Model 1	Model 2	Model 3
Age (year)	0.171	0.061	0.056	0.096
Sex (M/F)	0.001	0.516	0.222	0.058
Systolic blood pressure (mmHg)	0.060	0.006	0.001	0.002
Diastolic blood pressure (mmHg)	0.355	0.654	0.59	0.385
Pulse rate (PR, beats/per minute)	0.498	0.98	0.805	0.843
Body Mass Index (BMI, kg/m^2^)	0.319	0.921	0.059	0.506
*Smoking status (n, SD/%)*				
Smoking index (SI)	0.028	0.306	0.191	0.105
length of stay (LOS)	0.606	0.191	0.222	0.096
*Complications*				
Diabetes mellitus (DM)	0.354	0.608	0.523	0.791
Coronary atherosclerotic heart disease (CHD)	< 0.001	0.072	0.057	0.136
Hypertension	0.008	0.263	0.185	0.263
History of cerebral infarction	0.004	0.056	0.060	0.089
Procalcitonin (ng/mL)	0.752	0.031	0.03	0.067
C-reactive protein (CRP, mg/L)	0.092	< 0.001	< 0.001	< 0.001
Interleukin 6 (IL-6, pg/mL)	0.700	0.123	0.122	0.122
Hemoglobin (g/L)	0.147	0.31	0.287	0.304
White blood cell count (WBC, × 10^9^/mL)	0.013	0.008	0.002	0.006
Red blood cell count (RBC, × 10^12^ /mL)	0.400	0.035	0.03	0.045
Red cell distribution with (RDW, %)	< 0.001	< 0.001	< 0.001	< 0.001
Mean platelet volume (MPV, fL)	0.065	0.938	0.747	0.872
Fibrinogen degradation products (FDP, μg/mL)	0.499	0.522	0.973	0.94
D-Dimer (DD, ng/mL)	0.743	0.133	0.491	0.205
Albumin (ALB, g/L)	0.513	0.553	0.584	0.454
Abnormal albumin globulin ratio (A/G)	0.091	0.215	0.218	0.207
Triglyceride (TG, mmol/L)	0.410	0.941	0.994	0.861
Total cholesterol (TC, mmol/L)	0.842	0.271	0.196	0.233
High density lipoprotein cholesterol (HDL-C, mmol/L)	0.994	0.115	0.102	0.165
Low density lipoprotein cholesterol (LDL-C, mmol/L)	0.841	0.063	0.058	0.052
Total bilirubin (TBIL, μmol/L)	< 0.001	0.028	0.023	0.025
N terminal pro B type natriuretic peptide (NT-proBNP, pg/ml)	< 0.001	< 0.001	< 0.001	< 0.001

Model 1 matched variables; Sex, age and BMI. Model 2 added SI to model 1. Model 3 Added underlying diseases to model 2 (DM, CHD, hypertension, history of cerebral infarction)

### Logistic regression analysis

RDW, TBIL and NT-proBNP were stratified, and then multivariate logistic regression was performed to investigate associations between biomarkers levels and the prediction of AECOPD-PH. The results supported elevated NT-proBNP and TBIL might be significantly associated with AECOPD-PH, excepted for RDW (Table 3). Significant predictive ability was found for AECOPD-PH with TBIL < 13.8 μmol/L (OR = 0.598, 95%CI: 0.413–0.864), TBIL at the range of 13.8–16.5 μmol/L (OR = 0.485, 95%CI: 0.280–0.841) and NT-proBNP > 1165 pg/mL (OR = 0.559, 95%CI: 0.338–0.926), NT-proBNP at the range of 271-1165 pg/mL (OR = 0.293, 95%CI: 0.184–0.467). In the analysis of logistic, there was no association in the elevated RDW with low/moderately elevated RDW value (OR = 0.806, 95%CI: 0.547–1.186 vs OR = 0.933, 95%CI: 0.587–1.481). The analysis showed that the elevated RDW might not be associated with AECOPD-PH.

**Table 3:** Multivariate logistic regression analysis model of biomarkers

Values	Group 2	Group 3	B	S.E	Wald	P value	OR	OR (95%CI)	
	n = 688	n = 206						Lower	Upper
NT-proBNP > 1165 pg/mL	61	48	− 0.581	0.258	5.092	0.024	0.559	0.338	0.926
NT-proBNP 271–1165 pg/mL	160	57	− 1.228	0.238	26.581	< 0.001	0.293	0.184	0.467
NT-proBNP < 271 pg/mL	497	101			29.444	< 0.001			
TBIL > 16.5 μmol/L	142	72	− 0.723	0.28	6.644	0.01	0.485	0.28	0.841
TBIL13.8–16.5 μmol/L	96	24	− 0.515	0.188	7.471	0.006	0.598	0.413	0.864
TBIL < 13.8 μmol/L	450	110			9.949	0.007			
RDW > 14.6%	171	73	− 0.07	0.236	0.087	0.767	0.933	0.587	1.481
RDW13.6–14.6%	139	42	− 0.216	0.197	1.202	0.273	0.806	0.547	1.186
RDW < 13.6%	378	91			1.294	0.524			
Constant			0.201	0.239	0.711	0.399	1.223		

### ROC analysis of blood biomarkers

**Fig. 4:**
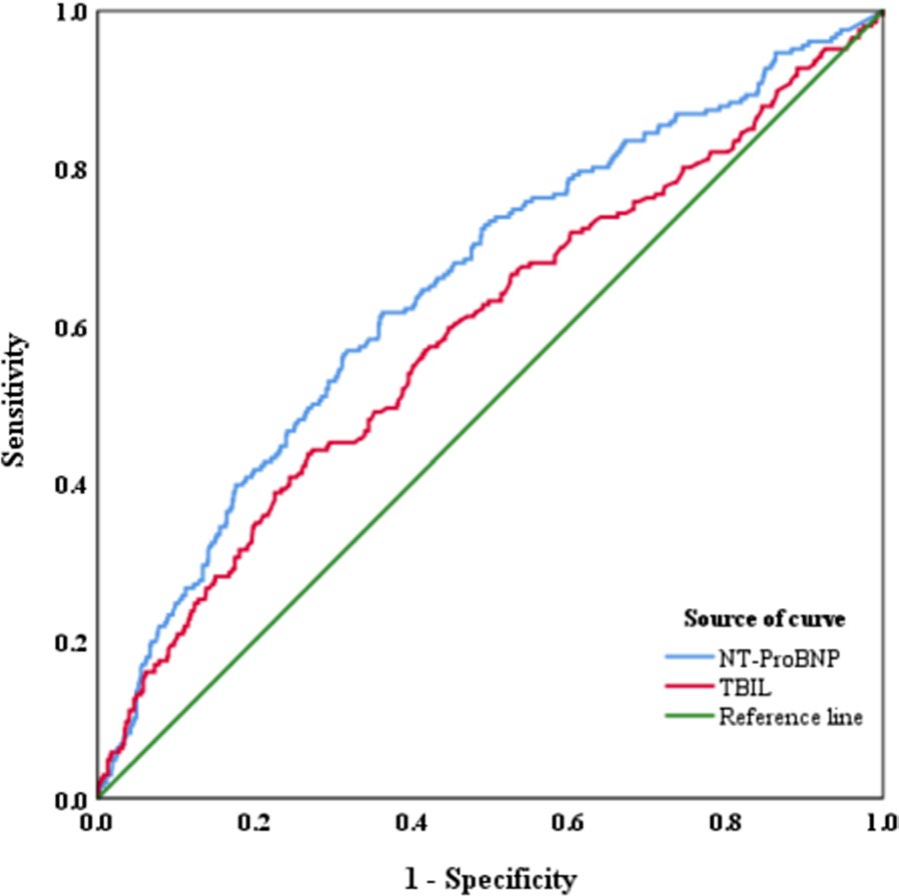
ROC analysis of TBIL and NT-proBNP

We calculated the ROC curve generated by significant indicators from previous analysis. The variables showed predictive ability for AECOPD-PH. The validity of NT-ProBNP in predicting AECOPD-PH was significant (P < 0.001). The AUC of NT-proBNP (0.651) was higher than TBIL (0.590). The optimal threshold of NT-proBNP (175.14 pg/mL) corresponded to predict whether AECOPD patients complicated with PH. The cut-off value of NT-proBNP was 175.14 pg/mL (sensitivity: 0.617, specificity: 0.638) in predicting AECOPD-PH (P < 0.001). The cut-off value of TBIL was 15.07 μmol/L (sensitivity: 0.437, specificity: 0.732), the AUC was 0.590 (P < 0.001) (Table 4 and Fig. 4).

**Table 4:** Receiver operating characteristic of markers

Marker	AUC	P value	Cut-off value	Sensitivity	Specificity	Youden index
NT-proBNP (pg/ml)	0.651	< 0.001	175.14	0.617	0.638	0.255
TBIL (μmol/L)	0.590	< 0.001	15.07	0.437	0.732	0.169

### Rank correlation analysis

Rank correlation was performed to explore whether there was a correlation between high levels of NT-ProBNP (≥ 175.14 pg/ml) / TBIL (≥ 15.07 μmol/L) and PASP value. Detecting the correlation between PH severity (based on PASP value) and NT-ProBNP / TBIL, a weak correlation of NT-ProBNP was reported in AECOPD-PH group (*r*_*s*_ = 0.299, P = 0.001). However, the correlation between TBIL and PASP was no significantly different (*r*_*s*_ = 0.173, P = 0.105) (Table 5). In view of the above study, NT-ProBNP, TBIL and RDW levels were surefooted biomarkers to help to identify AECOPD-PH patients. Correlation analysis showed that TBIL and RDW were correlated with NT-ProBNP levels in a weak correlation (RDW was 0.359 and TBIL was 0.238, P < 0.001) (Table 6).

**Table 5:** Rank correlation analysis

Biomarkers	n	*r*_*s*_	P value
TBIL	89	0.173	0.105
NT-proBNP	127	0.299	0.001

When the confidence (bilateral) was 0.05, the correlation was significant

**Table 6:** Correlation analysis of NT-proBNP with RDW / TBIL

Biomarkers	*r*_*s*_	P value
RDW	0.359	< 0.001
TBIL	0.238	< 0.001

## Discussion

This analysis is a large retrospective case-controlled study to examine the role of NT-proBNP in AECOPD-PH. Our findings suggest that NT-proBNP has a diagnostic efficacy to predict morbidity in patients with AECOPD-PH and 175.14 pg/mL is a predictive NT-proBNP threshold. And NT-proBNP has a weak correlation with severity of PH with AECOPD.

COPD is a common chronic disease, and its incidence has gradually increased recently [12]. PH is a common complication of COPD, which seriously affects the prognosis and quality of life of patients [13]. AECOPD-PH can cause death in severe cases [14]. The prevalence of PH in AECOPD depends on the severity of the disease and the definition of PH. Pulmonary hypertension is a common complication of COPD, and the presence of pulmonary hypertension is associated with shorter survival and more frequent exacerbations [15]. Although pulmonary hypertension is usually found in patients with advanced COPD, changes in pulmonary blood vessels have been observed in non-hypoxic, mild COPD subjects and in smokers with normal lung function. Changes in vascular structure may progress and lead to pulmonary hypertension, a complication of disease that occurs in more than 50% of patients with advanced COPD [15].

In our research, we conducted a preliminary statistical analysis of demographic data and some biomarkers, and found that age, gender, complications, smoking and other indicators were statistically significant, in order to eliminate the influence of these confounding factors, PSM (caliper value = 0.05, 1:1 matched) was used to analyze data, NT-proBNP, TBIL and RDW were significantly different in the three groups (P < 0.05), and these models did indicate that the results were stable and reliable. Then NT-proBNP, TBIL and RDW were performed by multivariate logistic regression analysis to test the predicting AECOPD-PH relevance, NT-proBNP and TBIL had statistical significance (P < 0.05). Then ROC curve was used to examined the diagnostic efficacy of NT-proBNP and TBIL, and found that NT-proBNP was the most suitable biomarker for diagnosing AECOPD-PH (AUC = 0.651). Finally, NT-proBNP had a weak correlation with severity of AECOPD-PH.

PH increases natriuretic peptide secretion in the right-sided heart chambers. Gene expression of NT-proBNP is up-regulated in the right atria with increased pressures, disruption of the natriuretic peptide receptor NPR-A worsens hypoxia-induced PH [16]. NT-proBNP is typically influenced by age, gender, and obesity in addition to renal function [17], in our research, first, we excluded the patients with renal insufficiency, then data analysis indicated age and gender indeed had a significant difference between COPD group and AECOD-PH group, however, BMI had no significant difference (P > 0.05), it might be associated with low distribution of obesity in China. We and other studies revealed low NT-proBNP levels in stable COPD patients [18].

In the absence of a significant left heart disease, NT-proBNP serves as a biomarker of an increased workload of the right heart originating from pulmonary arterial hypertension [19, 20]. We did not have the opportunity to assess the NT-proBNP levels during periods of stable COPD, since data have shown these values to be significantly lower than during AECOPD group or AECOPD-PH group. The results showed that elevated NT-proBNP levels predicted morbidity rather than severity of AECOPD-PH. First, we excluded chronic cardiac insufficiency in all enroll patients, NT-proBNP elevation was a result of AECOPD-PH, different statistical models were performed to draw a reliable and stable conclusion of the demographic data and different biomarkers in AECOPD and AECOPD-PH groups, we supported that NT-proBNP could predict the morbidity of AECOPD-PH, accordingly, in our study, elevated NT-proBNP levels (≥ 175.14 pg/mL) had a more significant predictive value than TBIL (AUC = 0.651 vs AUC = 0.590), which was diagnosed with sensitivity (61.7%) and specificity (63.8%). Based on the PASP values of echocardiography (it allows an estimation of the pulmonary artery pressure and gives an overall impression of the right and left heart deformities and function), the AECOPD-PH group was further divided into several subgroups (mild, moderate and severe). However, elevated NT-proBNP had a weak correlation with severity of AECOPD-PH (*r*_*s*_ = 0.299, P = 0.001). Macchia et al. supported that the assessment of NT-proBNP was useful for the detection of ventricular dysfunction in patients with COPD. A cut-off value of 160 pg/mL increased > tenfold the probability of finding ventricular dysfunction with echocardiography, it is basically consistent with our ROC results. For AECOPD patients, NT-proBNP measurement may help identify patients with PH. This association has not been replicated in other studies with larger COPD populations, making the prior observation a potential consequence of a smaller sample size [21, 22]. We were unable to assess the association of NT-proBNP independently in a multivariate analysis due to the low in-hospital mortality rate in our population.

For many years, RDW was almost exclusively used for the differential diagnosis of anemia. Recently it was increasingly investigated as a negative prognostic factor in variety of acute and chronic medical conditions, such as cardiovascular disease, community-acquired pneumonia [23]. Several studies showed that increased RDW is associated with disease severity and long-term mortality in COPD patients [24, 25]. Ozgul et al. showed increased RDW values in COPD patients compared to controls as well as in smokers compared to nonsmokers [25–27]. In our study, unadjusted data and PSM models (calipers value = 0.05) all showed RDW indeed had a significant difference in AECOPD-PH group (P < 0.05). However, in logistic regression analysis, elevated RDW had no statistic difference compared with low/moderately elevated RDW value (P > 0.05). The results supported elevated RDW might not be associated with AECOPD-PH. It is rather unquestionable that biological and metabolic abnormalities associated with human disorders may also exert a considerable influence on RDW. Such as, short or critically short telomeres (i.e. the DNA–protein structures located at the ends of chromosomes) lead to cell senescence of hematopoietic progenitors, especially those of the erythroid lineage, thus leading to increased replicative stress and impaired maturation of the erythroid lineage [28]; Oxidative stress has a profound influence on erythrocyte homeostasis and survival [29]. Oxidative stress may be another underlying biological mechanism that may lead to increased RDW possibly through increased red cell turnover, thus contributing to the association between anisocytosis and human pathology; And inflammation is commonplace in most human disorders and since several proinflammatory cytokines inhibit synthesis or activity of erythropoietin [30], inflammation might, in fact, promote anisocytosis through impairment of iron metabolism and disruption of response erythropoietin, thus impairing erythrocyte maturation and causing immature erythrocytes to enter the blood flow. Inflammation can also lower erythrocyte survival, thus leading to a more mixed population of RBC volumes in the circulation [23]. Those might be the underlying pathogenesis between RDW and COPD.

Total bilirubin (TBIL) has been considered as a powerful endogenous antioxidant in recent years. A large epidemiologic study found bilirubin concentrations to be negatively associated with COPD incidence [31]. Apperley et al. draw a conclusion that bilirubin is inversely related to COPD disease severity and progression [32]. Our data analysis was similar to it, compared with TBIL < 13.8 μmol/L, TBIL13.8–16.5 μmol/L had a lower protective trend than TBIL > 16.5 μmol/L (OR = 0.485, 95%CI: 0.280–0.841 and OR = 0.598, 95%CI: 0.413–0.864, respectively). There is a strong biologic rationale as to why bilirubin may have a modulatory role on lung function in COPD. The biochemical production of bilirubin begins when heme is degraded to biliverdin by heme oxygenase, biliverdin is subsequently reduced to bilirubin by biliverdin reductase [33]. The inducible isoform of heme oxygenase, heme oxygenase-1 (HO-1), has been shown to be up-regulated by oxidative stress [34], and its expression is also increased by hypoxia. Within the lung, HO-1 is expressed in type 2 pneumocytes and alveolar macrophages [35]. In our study, TBIL had a lower predictive value than NT-proBNP, and it had no significant difference in correlation with AECOPD-PH severity (P > 0.05).

In conclusion, we suggest that NT-proBNP has strongly diagnostic efficacy to predict morbidity in patients with AECOPD-PH and 175.14 pg/ mL is a predictive NT-proBNP threshold. And NT-proBNP has a weak correlation with severity of PH with COPD. COPD complied with PH was not a common disease; we have tried our best to collect as much sample size as possible.

Our study also has several limitations, such as a retrospective, single‑center design and the lack of a healthy control group. In our population and the mixture of patients with chronic COPD may also have biased this observation [36]. Most regretfully, since this is a large retrospective case-controlled study, few patients were eventually included in the log-rank, and we cannot make statistics on the prognosis of NT-proBNP in predicting AECOPD-PH.

## Conclusions

NT-proBNP has a diagnostic efficacy in AECOPD complicated with PH and NT-proBNP has a weak correlation with severity of PH with AECOPD.

## Data Availability

The datasets used and/or analysed during the current study are available from the corresponding author on reasonable request.

## Abbreviations

COPD: Chronic obstructive pulmonary disease
AECOPD: Acute exacerbation of chronic obstructive pulmonary disease
PH: Pulmonary arterial hypertension
NT-proBNP: N-terminal B-type natriuretic peptide precursor
TBIL: Total bilirubin
RDW: Width of Erythrocyte volume distribution
